# Development of a Questionnaire to Measure the Perceived Injustice of People Who Have Experienced Violence in War and Conflict Areas: Perceived Injustice Questionnaire (PIQ)

**DOI:** 10.3390/ijerph182312357

**Published:** 2021-11-24

**Authors:** Johanna Christina Neumann, Thomas Berger, Jan Ilhan Kizilhan

**Affiliations:** 1 Institute of Transcultural Health Science, Baden-Wuerttemberg Cooperative State University, 78054 Villingen-Schwenningen, Germany; Jan.Kizilhan@dhbw-vs.de; 2 Department of Clinical Psychology, Eberhard Karls University of Tübingen, 72074 Tübingen, Germany; 3 Department of Clinical Psychology and Psychotherapy, University of Bern, 3012 Bern, Switzerland; thomas.berger@psy.unibe.ch; 4 Institute for Psychotherapy and Psychotraumtology, University of Duhok, Duhok 42001, Iraq; 5 Transcultural Psychosomatic Department, MediClin, 78166 Donaueschingen, Germany

**Keywords:** injustice, trauma, war, crisis, PTSD, psychotherapy, inventory, diagnostics, justice, violence

## Abstract

Objectives: The primary aim of this research was to develop a questionnaire that assesses perceived injustice among survivors of war and trauma in conflict areas and to evaluate its psychometric properties. This paper presents the first preliminary validation. Furthermore, the assumption that the general perception of injustice correlates with one’s own experiences of injustice and violence was tested. Methods: The 24-item Perceived Injustice Questionnaire (PIQ) was administered partly online and partly in a paper–pencil version to 89 students of the University of Dohuk in Northern Iraq, an area that has been affected by crisis and war for many years. Principal component analysis was used for factor extraction and internal consistency was determined. The Mann–Whitney-U test was used to calculate the group differences between people with and without experience of physical violence and strong experiences of injustice because Kolmogorov–Smirnov tests showed that the data are not normally distributed. Results: Principal component analysis yielded a four-component solution with eigenvalues being the greater one. Cronbach’s alpha for each scale was acceptable to satisfactory. Significant results of the Mann–Whitney U tests supported our assumptions of between-group differences on each of the subscales (emotional and cognitive consequences, injustice perception, injustice experience, revenge, and forgiveness). Discussion: The findings of this study support the construct validity and the reliability of the PIQ. For this reason, it can be seen as a useful addition to the psychological assessment in psychotherapeutic settings of survivors of war and violence. In conclusion, and based on the PIQ, we suggest the development of a new set of therapy modules with worksheets, focusing on the perception, dealing, and understanding of feeling of injustice as an addition to the existing trauma therapy manual for therapy in war and conflict areas.

## 1. Introduction

People perceive different actions as unjust and react differently to injustice experiences [1]. This subjective perception of injustice can determine not only their actions but also their mental health. For example, research on people suffering from pain after traumatic accidents has shown that people who subjectively perceive their situation as less just feel pain for a longer time and more strongly [2,3,4,5].

Traumatic war experiences are often followed by psychological consequences such as post-traumatic stress disorder (PTSD) or depression [6]. Ten percent of people who had to flee their homeland due to natural disasters or persecution suffer from PTSD. Five percent of people who had experienced the same situation suffer from depression [7]. Rates are higher among survivors of rape, military action, captivity, internment for ethnic or political reasons, or genocide [6]. Researchers found a prevalence rate for PTSD of 56% for female survivors of the Rwandan genocide [8] and a prevalence rate for depression of 80% for women affected by sexual violence in former Yugoslavia [9]. For Yazidi women who had survived the ISIS genocide and captivity in 2014, the prevalence rate for PTSD (58%) and depression (55%) was found to be very high even after five years [10]. It has been shown that people who experience direct man-made violence, such as survivors of rape or captivity, have a significantly higher risk of developing mental disorders as a consequence of the traumatic experience than people who experience natural disasters [7]. Additionally, people who were deliberately harmed by others, such as survivors of sexual trauma or of mass conflicts, generally experience situations as more unjust than those who were harmed unintentionally, such as survivors of natural disasters [11].

Studies on survivors of the violent conflicts in Rwanda and Timor-Leste suggest a connection between traumatic experiences, post-traumatic stress disorder (PTSD), and the subjective perception of injustice [12,13]. However, not all studies which have focused on post-conflict areas could find this connection [14]. To better understand the impact of injustice experiences on mental health, it is necessary to validly assess experiences of injustice with suitable instruments. Yet, all of the common inventories to survey the perception of injustice have been developed in Western societies [15,16,17,18]. Furthermore, most evaluations of a sense of justice were conducted in Western cultures up to this point. Frequently, the focus was on concepts such as social injustice, fair distribution of goods, or justice sensitivity (e.g., Justice Sensitivity Inventory; [17]) or the questionnaire was developed to determine the belief in a just world [15], a concept that was developed based on a Western idea of justice. In collaboration with non-Western experts and participants, we wanted to develop an inventory that might capture the perception of justice from a more diverse perspective to be able to be applied across multiple cultures.

This shows that not many scales are measuring the justice perception after traumatizing war experiences. Most studies in that field developed and used specific questionnaires which only referred to the target group or the conflict concerned [14]; Pham et al., 2004b). Research has shown that the perception of injustice is likely to increase through situations that are characterized by basic human rights violations [2]. For these reasons, it seems necessary to have an inventory that can be widely used to approach people’s perception of injustice after experiences of severe human rights violations.

Data collected with such an inventory can later become a support for claims of legal reparations for the survivors of such injustice experiences. Medical doctors and psychologists agree that some form of justice must be achieved to mentally process what has been experienced [19]. “No healing without justice” says Dr. Mukwege, Nobel Peace Prize laureate 2018, about his work with women and children who survived sexual violence in Eastern Congo. Referring to the victims of ISIS in Northern Iraq, psychotraumatologist Professor Kizilhan and Nadia Murad, the other 2018 Peace Prize laureate, emphasize that the psychological wounds of women can only be healed if they are also given legal justice [19]. Yet, legal processes and achievements for reparations are often not given or only hard to achieve after war and human right violations in crises areas. For this reason, it is necessary to address the topic of injustice experiences also in a psychotherapeutic setting.

We aimed to develop a new inventory that collects information on how individuals in critical areas categorize potentially unjust experiences, whether those affect their perception of justice in general, and how they cope with that perception. This is particularly relevant as it is assumed that one’s perception of injustice has an impact on various mental health conditions such as depression, anxiety, somatoform disorder, and PTSD [4,5,12]. Trauma increases the likelihood of developing PTSD and or depression, but the increased perception of injustice is most likely an additional contributor, according to research [12,13]. One explanation for this would be the violation of the belief in a just world (BJW); the belief that people get what they deserve and deserve what they get [20,21]. In order to maintain this belief despite the experiences of injustice, depressive thinking patterns can be developed such as self-blame [22]. Furthermore, studies with torture survivors show that the loss of the previously existing just world view can lead to distrust and sensitivity towards injustice in general being maintained and can manifest itself in depressive symptoms [23].

Moreover, we see clinical and research benefits in an instrument that explores injustice perceptions and their relevance for psychotherapy. We planned an inventory that can clarify the individual needs for trauma therapy and specify our understanding of the correlation between injustice perception and mental health in this vulnerable group. A better planning and organization of psychotherapeutic treatment necessitates an inventory that explores the individual perceptions of justice and the importance for psychotherapy. As we could not find an instrument which meets the necessary criteria, a new instrument was developed and tested.

Studies have shown that people who experience more violence and more potentially traumatic events are more likely to develop psychiatric disorders like depression or PTSD (6). We developed this questionnaire under the assumption that the same is true for the perception of injustice, meaning that people who experience more violence or other unjust treatment, would report a stronger sense of injustice. Leading to more explicit responses in each scale of the PIQ in comparison with those who report less violence and injustice experiences.

## 2. Methods

### 2.1. Development of the PIQ

First, an unsystematic, preliminary literature research was used to determine questions for the focus groups. Keywords such as “perceived injustice”, “injustice in psychotherapy”, “trauma and injustice”, “perceived injustice”, “justice and mental health”, “transitional justice”, and “restorative justice” were searched for in various combinations. In connection to a literature review, we aimed to sufficiently encompass the concept of perceived injustice in order to adapt it to the chosen target group accordingly. The existing justice and injustice inventories were reviewed.

Between May 2019 and October 2019, we conducted several focus groups and interviews with refugees and survivors of war and prosecution in Germany and Iraq. We aimed to identify feelings of injustice, an understanding of justice, and coping mechanisms. The topic was discussed with experts in psychotraumatology, legal practice and reparations, and social work. Experts were contacted in Germany, Turkey, Israel, and Iraq. Detailed information on the people interviewed and the setting can be found in Appendix A and in [24].

**Figure A1:**
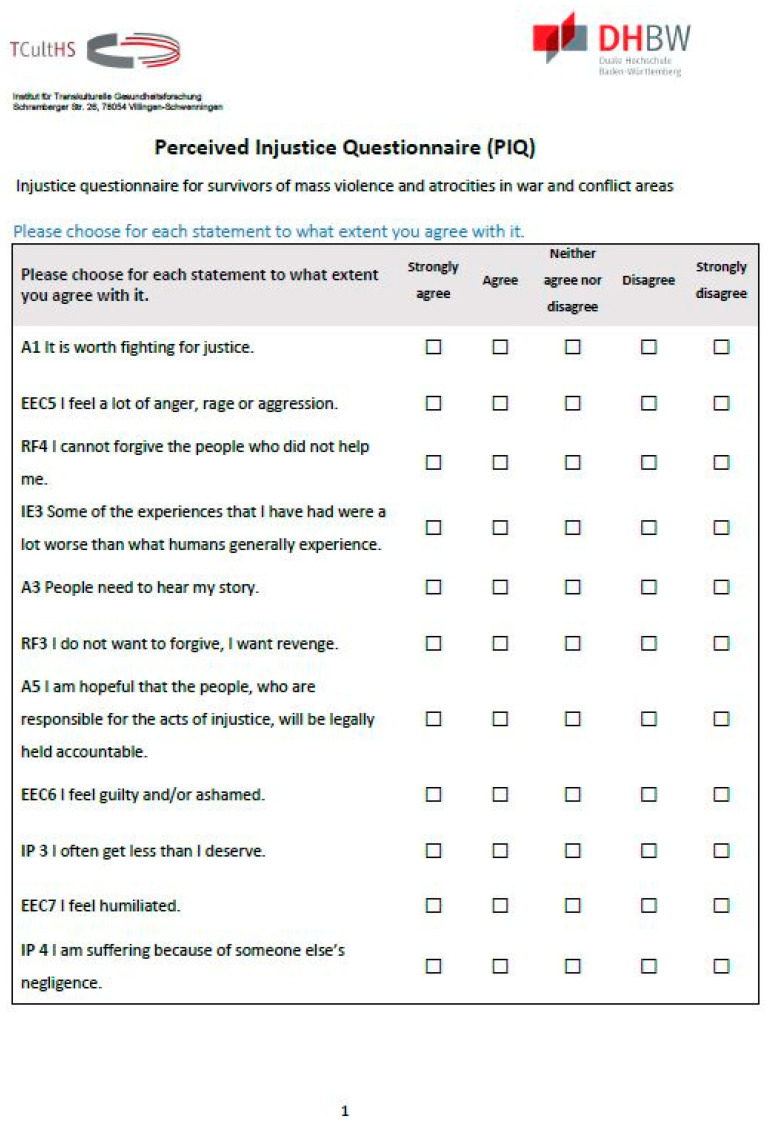
The PIQ, page 1.

**Figure A2:**
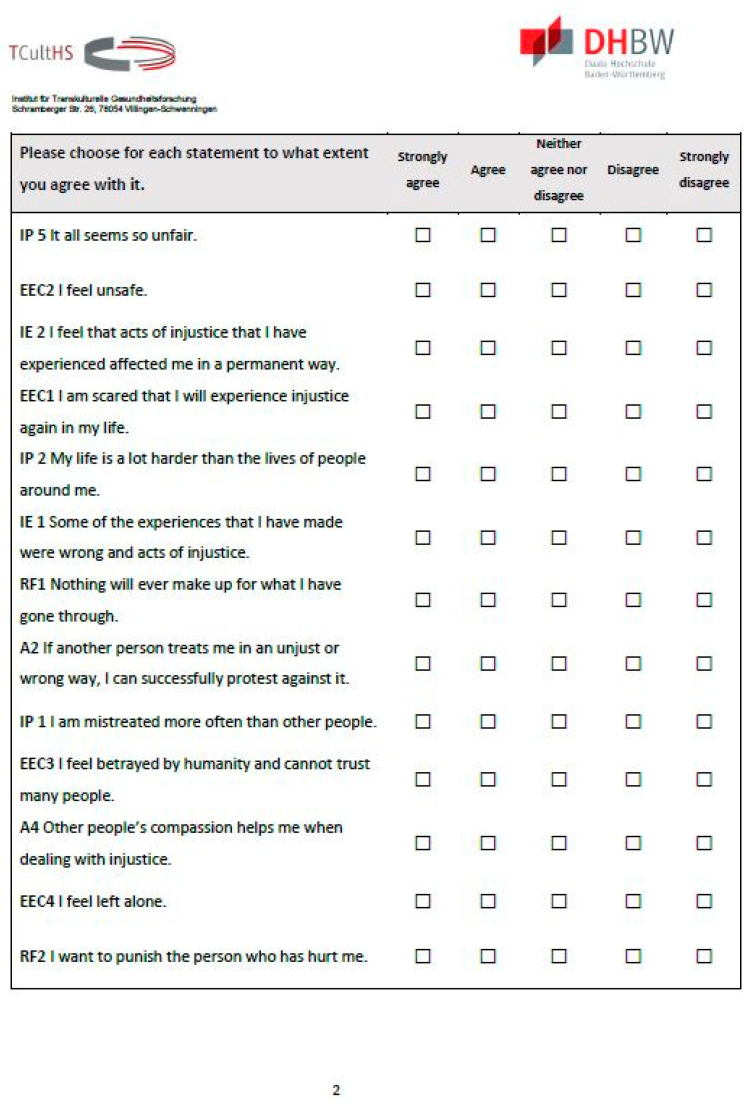
The PIQ, page 2.

**Figure A3:**
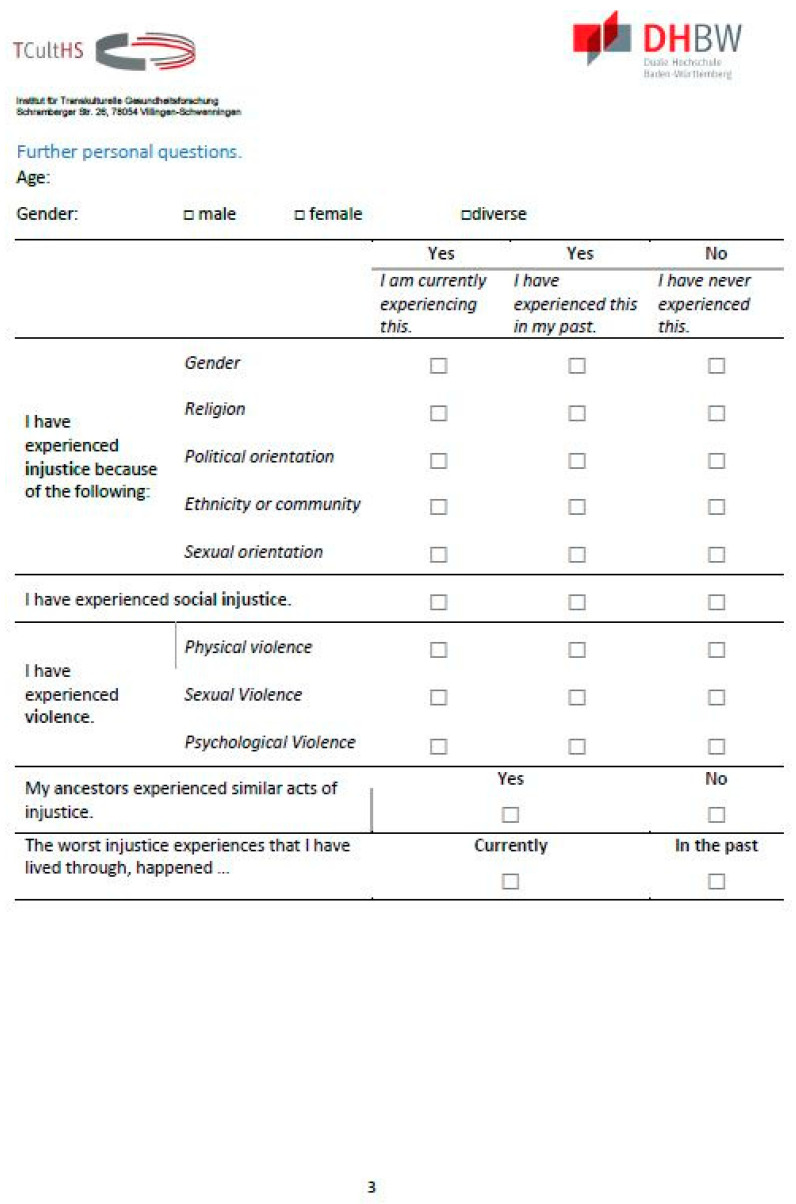
The PIQ, page 3.

Based on the interviews with affected individuals and experts, focus groups, literature research, and existing questionnaires, we developed a first preliminary item pool. Repetitive items were then deleted and the wording was adjusted. All these steps were taken in consultation with an expert in psychotraumatology. This process resulted in 27 items for the questionnaire and further questions on each person (Appendix B Figure A1, Figure A2 and Figure A3).

The further questions inquire about demographics and whether injustice was experienced and what kind of injustice this was, distinguishing between injustice experiences based on gender, religion, ethnicity, political, or sexual orientation, social injustice experiences, and experiences of physical, sexual, or psychological violence (Appendix B Figure A1, Figure A2 and Figure A3). In addition, it is recorded whether the experiences were made in the past or are still taking place. In addition, the ancestors’ experiences of injustice are also examined, as their emotional and possibly traumatic consequences can be passed on through generations [25]. These questions are essential for both therapy and research. Persistent experiences of violence and injustice can affect the outcome of therapy. In connection with that, knowledge of past experiences can provide clues that can be used to find the right interventions.

All items were administered in English. As there are not enough clinical questionnaires translated and validated into Kurdish or Arabic, it is not uncommon to use English-language inventories in the planned areas of application.

### 2.2. Study Population

For the first validation phase, the preliminary items were presented to and completed by 89 students at the University of Dohuk, Northern Iraq. Without a power analysis, the best accessible sample was used for this first preliminary validation. The geographical area of Northern Iraq is part of the Kurdistan region (KRI), an autonomous region recognized by the constitution of Iraq. The population in this region is characterized by its ethnic diversity. Muslims coexist with religious minorities such as the Yazidis and some Christians. In August 2014, troops of the self-proclaimed Islamic State (ISIS) conquered areas of Northern Iraq, using extreme brutality against religious minorities such as the Yazidis. Around the city of Dohuk, there are currently 20 refugee camps for about 600,000 refugees including 450,000 Yezidi and 50,000 Christians. Due to the ongoing war in the neighboring country of Syria, new people are still arriving (https://www.igfm.de/so-hilft-die-igfm-fluechtlingen-im-nordirak/, retrieved 29 October 2020). Although our sample are all students at the university, they were born and raised in the conflicted area of Northern Iraq, lived through the Iraq war and part of them are members of the Yazidi or Christian minority. They are survivors of an ongoing crisis, although they are not diagnosed with PTSD.

A total of 38 students (42.69%) completed the questionnaire online. At that time, all participants were students at the *Institute for Psychotherapy and Psychotraumatology* at the University of Dohuk. The other 50 students (57.31%) completed a paper–pencil version of the same questionnaire. At that time, all participants were students at other faculties at the University of Dohuk. Both questionnaire versions allowed participants to write comments after every scale to note difficulties or suggestions for improvement.

A total of 52students (58%) were male and 36 students (42%) were female; the average age was 26 years (SD = 6.24, between 19 and 54). Out of all interviewed students, 67.4% (*n* = 60) strongly agreed or agreed with the item “Some of my experiences were wrong and acts of injustice”.

Almost all of the students (*n* = 51) who completed the paper–pencil version agreed or strongly agreed with either “I think I have experienced injustice because of my religion” and/or to “I think I have experienced injustice because of the community or ethnicity that I belong to”. Furthermore, many (*n* = 49) responded that their ancestors had experienced similar acts of injustice before them. A total of 28.1% (*n* = 51, *n* = 22) of participants agreed or strongly agreed that they had experienced physical violence.

Students who completed the online version could not provide the same detailed information about their person. Yet, it can be assumed that most of them would have answered the same way, as all of the participants belong to the Kurdish minority in Iraq, even though their religion (Muslim, Yazidi, and Christian) differs.

### 2.3. Methods of Analysis

Principal component analysis (PCA) was used for factor extraction and internal consistency was determined. Internal consistency is a widely used measure of the reliability for a psychiatric instrument. It refers to the extent to which a scale measures a common underlying construct. It can be assessed by Cronbachs’s alpha, a correlation statistic proposed by Cronbach [26]. The Kaiser–Meyer–Olkin score [27,28] and Barlett’s test of sphericity were used.

To get a first simple impression of whether there are differences between two groups, we artificially divided the Likert scale into two groups (“yes” and “no”). We divided the sample according to their demographic responses with regard to their experiences of violence and injustice. We used a Kolmogorov–Smirnov to test whether the data followed a normal distribution. Since this was not shown, Mann–Whitney U tests were used to calculate the group differences between people with and without experience of physical violence and strong experiences of injustice.

## 3. Results

### 3.1. Principal Component Analysis

We performed a PCA to extract the most important independent factors. A Kaiser–Meyer–Olkin score of 0.78 confirmed the adequacy of the data for this analysis, and Bartlett’s test of sphericity was significant (*p* < 0.001), indicating that correlations between items were sufficiently large for performing a PCA. Only factors with eigenvalues ≥ 1 were considered [27,29].

Examination of Kaiser’s criteria and the scree plot yielded empirical justification for retaining four factors with eigenvalues exceeding 1, which accounted for 69.78% of the total variance. Among the factor solutions, the Varimax-rotated four-factor solution yielded the most interpretable solution, and most items loaded highly on only one of the four factors. These factors were called “emotional and cognitive consequences” (EEC), “injustice perception” (IP), “injustice experiences” (IE), and “revenge and forgiveness” (RF). Results of the PCA can be found in Table 1. For content reasons, items IE 3, IP 4, and RF 1 were not assigned to the proposed factors. Instead, they were left in the original assigned subscale. The discriminatory power (r_it_) for the items in each scale fluctuated between r_it_ ≤ 0.487 and r_it_ ≤ 0.808. According to Fisseni’s [30] guidelines, this can be considered high.

### 3.2. Reliability

The reliability of all items was examined with regard to their originally assigned subscale. Items whose reliability in the originally assigned scale was <0.5 were excluded. Table 2 shows the reliabilities using Cronbach’s alpha, and mean values for each scale, which were acceptable to satisfactory.

### 3.3. Between-Group Differences (Mann–Whitney U Tests)

The differences between the groups were calculated with the use of Mann–Whitney U tests because the distributions differed between both groups, Kolmogorov–Smirnov *p* < 0.05. The significance level for all calculations was alpha = 0.05. The groups were divided according to answers to the questions about experiences of physical violence and injustice.

For all scales, ECC, IP, IE, and RF, there were significant differences between people with physical violence experiences and injustice experiences scored significantly higher than people who did not report such experiences. The details are listed in Table 3.

There were also significant differences in the total score. For the comparison between injustice experiences or no such experiences, Z = −4.38, U = 168, *p* < 0.001, and for the differences between people with and without physical violence, Z = −4.51, U = 70.50, *p* < 0.001.

Furthermore, the differences between these two groups with respect to their answers in the additional items were calculated. People who reported experiencing violence and people who reported other injustices scored higher in the items A3 and A4 than the other participants. There were no significant differences in item A1, A2, and A5, however it stands out that all groups on average answered with four or higher to the question “It is worth fighting for justice”. For more details see Table 4.

### 3.4. Description of the PIQ

The PIQ consists of 18 Likert scale items (Appendix B Figure A1, Figure A2 and Figure A3) in four scales plus five additional items. Excel was used to randomize the order of all these items. The items for the subscales “emotional and cognitive consequences” (EEC), “injustice perception” (IP), “injustice experiences” (IE), “revenge and forgiveness” (RF), and the additional items can be answered on a Likert scale of 1–5, where the value 1 corresponds to the statement “strongly agree” and 5 to “strongly disagree.” For the scoring see Appendix C.

## 4. Discussion

This work aimed to develop an instrument that can be applied to assess the individual perceptions of injustice experiences and their emotional and cognitive consequences in survivors of war and mass violence. Studies have shown a moderate to strong correlation between perceived injustice and depression [31]. Until now, the questionnaires used frequently referred specifically to one event or a specific study or assessed not only man-made injustice but also non-man-made disasters such as natural catastrophes or unintentional disasters, e.g., accidents.

However, the perception of justice is not mainly a mental construction [4] but is most often based on a number of justice violations, especially in the case of human rights violations. Therefore, the restoration of justice in post-war and crisis areas has to occur across many disciplines—on a judicial level, through medical support, social and political changes, but also on an individual, psychological level [19]. In order for this to be realized, stabilization and subsequent trauma therapy are necessary. The PIQ is meant to support this psychotherapeutic process.

We generated a first preliminary item pool through interviews and focus groups with refugees from different war and crisis areas. Cutting the items down and exploring them with a PCA we received four scales (emotional and cognitive consequences (ECC), injustice perception (IP), injustice experience (IE), and revenge and forgiveness (RF)). For content reasons, we left five additional items in an additional scale. Subsequently, these were tested with a sample of students in Dohuk, Northern Iraq.

A PCA supported our proposed subscales. Reliabilities for the subscales as well as the overall reliability were satisfactory. In our case, the present sample is very much homogenous and the instrument has been designed specifically for people in war and conflict areas. Northern Iraq can be considered as such a crisis region. The local and regional unrest has continued for many years. Most people come from families that have been affected by war, or other forms of violence for many years.

Convergent and discriminant validity of this novel instrument have yet to be evaluated. For the investigation of the discriminant validity, we propose, among others, to evaluate the correlations between our instrument (PIQ) and the bitterness questionnaire [16]. A correlation between the Injustice Experience Questionnaire [2] and our instrument can be used to examine its convergent validity because convergent validity states that tests having similar constructs should be highly correlated and are supposed to share some general parts of injustice experiences and at least be moderately correlated with each other [32].

For the use of the PIQ in therapy, five items that didn’t fit the analysis were incorporated because they access the coping strategies of people regarding their feelings of injustice. In a long-term perspective, these strategies can be used in psychotherapy to enable those who experienced traumatic and violent events to reduce negative feelings. Likewise, these strategies might help in shifting one’s injustice perception (IP) in a long-term perspective as well.

This inventory has been developed to improve the understanding of the perception of injustice and its impact on people’s health. We cannot help them regain a sense of justice or shift their focus, unless we know to what extent they perceive their own situation as wrong and unjust. This new inventory is intended to help trauma therapists in areas of war and crisis. It is meant to enable them to conduct a differentiated analysis of the problem and to determine the focus of the psychotherapeutic work. Based on this inventory, our research group is currently planning to develop a new set of therapy modules with worksheets which focus on the perception of, understanding of, and approach to feelings of injustice. The questionnaire can then be used as an introduction to the topic and as an assessment of its relevance.

### Limitations

However, psychometric properties such as reliability are dependent on the population and sample size and cannot be treated as fixed characteristics of the test [33,34]. Additionally, no test–retest validity has been assessed to date. This can be considered a limitation.

For this reason, the characteristics of this instrument can, at this point, neither be easily transferred to other populations nor to specific subgroups within this population. All participants in our example were healthy enough to be able to go to university and had an academic background, although many of them live in refugee camps themselves due to the ongoing crisis situation in this area.

The PIQ was formulated in the English language to be used as extensively as possible in a global context. Yet, the English language might be seen as a limitation because sensitive words such as justice or injustice cannot be translated in other languages as easily without changing their meaning. The sample knows English really well and have been studying in English since their bachelor’s degrees and are used to working with English textbooks, questionnaires, and literature because there isn’t much material in Arabic and even less in Kurdish. Furthermore, English is the only common language of the sample, as Arabic and Kurdish, as well as various Kurdish dialects, are spoken in the region, which are not understood and spoken by all. Once our inventory is further reviewed and somewhat established, the plan is to create and validate further translated versions.

Based on the literature research, it can be assumed that people who have experienced more physical violence, such as people with severe physical trauma (3), are likely to be more sensitive to perceived injustice than people without those experiences. In order to validate the content of the questionnaire, groups with and without experience of violence were therefore compared. Further, the results of the Mann–Whitney U tests give us first indications that there are large differences in the perception of justice depending on the experiences one has had in life.

However, we cannot yet see whether the perception of these events and justice directly affects or moderates mental health or not. For this, it is relevant to retest this inventory with, among others, people who have received a PTSD diagnosis due to their experiences.

Therefore, further validation studies should focus on more specific samples. For example, future studies should examine reliabilities for groups of individuals with and without diagnosed depression and PTSD or with both disorders, as these are some of the most common disorders in survivors of genocide, war, and mass violence [6]. Another idea is to perform a split-reliability assessment based on people’s sex, since trauma upon women is one of our research foci.

It is planned to further validate the PIQ in the outpatient clinic recently founded at the Institute for Psychotherapy and Psychotraumatology (IPP) at the University of Dohuk, Iraq. Thus, the validity of this instrument can be verified again for the intended target group.

## 5. Conclusions

With the PIQ, we have developed an inventory to determine both the relevance and perceptions of injustice among people in conflict areas. Since we have used an academic sample, further validation studies with a larger and more heterogeneous sample are planned.

## Figures and Tables

**Table 1:** Factor loads and discriminatory power (r_it_).

	Factor 1	Factor 2	Factor 3	Factor 4	r_it_
Emotional and cognitive consequences (ECC)					
Item ECC 1	0.775	0.343			0.740
Item ECC 2	0.836	0.306			0.712
Item ECC 3	0.846				0.744
Item ECC 4	0.731				0.740
Item ECC 5	0.523		0.503		0.633
Item ECC 6	0.685		0.336		0.607
Injustice perception (IP)					
Item IP 1		0.664	0.331		0.770
Item IP 2		0.779		0.341	0.808
Item IP 3		0.796			0.705
Item IP 4		0.531	0.688		0.666
Item IP 5		0.761	0.381		0.789
Injustice experience (IE)					
Item IE 1			0.668		0.525
Item IE 2			0.651		0.560
Item IE 3	0.596		0.381	0.350	0.529
Revenge and forgiveness (RF)					
Item RF 1	0.699		0.346	0.324	0.487
Item RF 2	0.317			0.654	0.649
Item RF 3				0.762	0.659
Item RF 4				0.669	0.668
Variance explained	25.338%	17.564%	13.710%	13.167%	

Note: factor loads ≥ 0.30 after Varimax rotation.

**Table 2:** Cronbach’s alpha and mean values for each subscale.

Total Sample (N = 89)	Amount of Items	Mean	Standard Deviation	Cronbachs Alpha
Injustice Experience (IE)	3	3.56	0.67	0.710
Injustice Perception (IP)	5	3.02	0.79	0.895
Emotional and cognitive consequences (ECC)	6	2.95	0.79	0.883
Revenge and Forgiveness (RF)	4	3.32	0.78	0.800

Overall Cronbach’s alpha = 0.929

**Table 3:** Mann–Whitney U test tables for mean values of scales.

			*n*	M	SD	Z	U	*p*	r ^c^
Emotional and cognitive consequences (ECC)	Violence experience	No	25	2.42	0.51	−4.37	77	<0.001 ***	0.63
		Yes ^a^	23	3.46	0.64				
		Total	48	2.92	0.78				
	Injustice experience	No	29	2.64	0.80	−2.77	505	0.006 **	0.30
		Yes ^b^	55	3.11	0.74				
		Total	84	2.95	0.79				
Injustice perception (IP)	Violence experience	No	26	3.11	0.40	−4.19	105.5	<0.001 ***	0.59
		Yes	25	3.66	0.50				
		Total	51	3.38	0.53				
	Injustice experience	No	22	2.58	0.62	−3.56	260	0.001 ***	0.42
		Yes	50	3.22	0.78				
		Total	72	3.02	0.79				
Injustice experience (IE)	Violence experience	No	26	3.27	0.49	−4.51	91	<0.001 ***	0.63
		Yes	25	4.04	0.49				
		Total	51	3.65	0.62				
	Injustice experience	No	29	2.87	0.44	−6.91	92.5	<0.001 ***	0.73
		Yes	60	3.89	0.49				
		Total	89	3.56	0.67				
Revenge and forgiveness (RF)	Violence experience	No	24	3.17	0.34	−4.95	52.5	<0.001 ***	0.71
		Yes	24	4.02	0.48				
		Total	48	3.59	0.60				
	Injustice experience	No	28	2.86	0.43	−4.78	265	<0.001 ***	0.53
		Yes	53	3.57	0.81				
		Total	81	3.32	0.78				

Note: ** *p* ≤ 0.01, *** *p* ≤ 0.001. ^a^ Violence experience “Yes”: people who answered “1: strongly agree” or “2: agree” to the question: “I have experienced physical violence.”. ^b^ Injustice experience “Yes”: people who answered “1: strongly agree” or “2: agree” to the question: “Some of my experiences were wrong and acts of injustice.” ^c^ Effect size measured via Pearson correlation coefficient $r=\left|Z\right|:\;\sqrt{N}$.

**Table 4:** Mann–Whitney U test tables of additional items.

			*n*	M	SD	Z	U	*p*	r ^a^
Item A1: “It is worth fighting for justice.”	Violence experience	No	26	4.35	0.63	−1.33	251.50	0.185	-
		Yes	24	4	0.93				
		Total	50	4.18	0.80				
	Injustice experience	No	29	3.79	0.94	−1.08	715.5	0.810	-
		Yes	57	3.93	1.12				
		Total	86	3.88	1.06				
Item A2: “If another person treats me in an unjust or wrong way, I can successfully protest against it.”	Violence experience	No	26	3.58	0.50	−2.32	210	0.210	-
		Yes	24	3.25	0.44				
		Total	50	3.42	0.50				
	Injustice experience	No	29	3.62	0.73	−1.37	688	0.170	-
		Yes	57	3.42	0.78				
		Total	88	3.49	0.76				
Item A3: “People need to hear my story.”	Violence experience	No	26	2.92	0.69	−3.98	120	≤0.001 ***	0.56
		Yes	24	3.83	0.70				
		Total	50	3.36	0.83				
	Injustice experience	No	29	2.59	0.95	−4.10	396	≤0.001 ***	0.44
		Yes	57	3.51	0.95				
		Total	86	3.2	1.04				
Item A4: “Other people’s compassion helps me when dealing with injustice”.	Violence experience	No	26	4.15	0.09	−2.89	185	0.004 **	0.41
		Yes	24	4.58	0.10				
		Total	50	4.36	0.07				
	Injustice experience	No	29	3.41	0.18	−3.24	499.50	0.001 ***	0.35
		Yes	57	4.09	0.12				
		Total	86	3.86	0.11				
Item A5: “I am hopeful that the people who are responsible for the acts of injustice will be legally held accountable.”	Violence experience	No	26	4.04	0.92	−8.08	273	0.419	-
		Yes	24	3.83	0.82				
		Total	50	3.94	0.87				
	Injustice experience	No	28	3.11	1.26	−1.28	667.50	0.202	-
		Yes	57	3.42	1.09				
		Total	85	3.32	0.12				

Note: ** *p* ≤ 0.01, *** *p* ≤ 0.001. For the mean values and all further evaluations, value 1 corresponds to the statement “strongly agree” and 5 to “strongly disagree”. ^a^ Effect size measured via Pearson correlation coefficient $r=\left|Z\right|:\;\sqrt{N}$.

## Data Availability

The data presented in this study are available on request from the corresponding author.

## Appendix A

### Appendix A.1. Detailed Information about Focus Groups and Original Interviews

#### Appendix A.1.1. Methods

- Focus groups and interviews. The survey with the questions for the face-to-face interviews were distributed to many different psychosocial institutions in Germany and through some international organizations around the world. Yet, the responses were little.
- Audio recording was possible only in one interview (14). In all focus groups, there was at least one person who did not wish to be recorded after being asked for permission.
- The institutions provided the interpreters. In two groups (1 + 4) they were non-sworn and came to Germany as refugees as well. In group 2, the attending psychotherapist and the social worker interpreted.
- The group discussions lasted between 45 to 70 min

(1) Focus group with men from the Gambia, who are diagnosed with PTSD. Focus discussion happened during their official stabilization group (refugio Stuttgart, Germany). (2) Focus group with patients of the transcultural department of a psychosomatic rehabilitation clinic, of whom some are diagnosed with PTSD (Donaueschingen, Germany). (3) Focus group with Yazidi women, part of their therapeutic group session, (PBV, Stuttgart, Germany). (4) Focus group with refugees in a German level A2 in Germany (Tuebingen, Germany); 11 students, male and female. (5) Focus group with students of the IPP in Dohuk, Northern Iraq. (6) Focus group/interviews with people in one refugee camp near Dohuk, Northern Iraq. (7) Interview with interpreter from group (1), who also fled from the Gambia. (8) Interview via survey with a Holocaust survivor (self-help home, Chicago, IL, USA). (9) Interview via survey, with a refugee from Syria (refugio VS; Germany). (10) Interview via survey, with a social worker from Burundi (zfd, Burundi). (11) Interview via survey, with a Yazidi woman from northern Iraq (PBV, Stuttgart, Germany). (12) Interview via survey, with a Kurdish woman from Iran (PBV, Stuttgart, Germany). (13) Interview via survey, with a man from Nigeria (PBV, Stuttgart, Germany). (14) Conversation/interview with Kurdish interpreter from group (3), who’s family is in northern Syria.

#### Appendix A.1.2. Overview of the Results

“What is justice?”: Everyone treated in the same way; everyone gets what they need, doesn’t have to be the exact same; dignity; all about the truth; treat people the way you want to be treated by them; an effort to be a good person; everybody lives in peace and lives well; getting the opportunities one needs; some are fighting for it, but it doesn’t exist; respect (other people(s), family, …); some countries will never get justice, others might [Syria–Germany]; Different kinds of injustice were mentioned: inequality in treatment in Germany or daily treatment (children, gender-based, …); inequality in the world and cruel wrongdoings such as ISIS and Boko Haram.

“Examples of injustice”: Innocent, but got charged by court; deportation; different treatment of refugees in Germany, depending on their origin (e.g., residence status; translators available); racism in Germany because of skin color or accent (at work, in school …); no human rights/torture/abuse…; Boko Haram; Libya: mistreatment, enslavement, torture; ISIS captivity, murder, and torture; not being able to see a doctor in Germany, having a translator; persecution of minorities; persecution of LGBT community; no/less rights for women; e.g., in court a men’s testimony counts as much as two women’s; dictatorship; not getting the opportunities one needs (no education, no attention, no love); lost freedom; bullying; no family reunification; broken promises; horrible conditions in the refugee camps; in Germany, mistreated by other refugees; differences in life standards between countries; world is just watching what is happening in Syria; ignorance and egoism in the large population of many countries.

“Emotional reactions”: Angry/aggression; stupor; poor sleep; self-pity; flashbacks; sadness and mourning; affective disorders; behavioral disorders; guilt; exhausted from trying to fight back; wish for (violent) revenge; suicide; cannot trust some people; fear.

“Strategies to deal with injustice”: Talk to each other; finding people you can trust and who support you and are by your side; respect each other; recognizing that there are also good, helpful, and just people; belief/prayer and hop/accepting that God will fix it; remaining a good person, even if it’s hard; accepting some unfair treatment; believing that everything hast to be, just stops one from being okay with one’s life; somehow you get through it; trying to change one’s mentality/taking action through campaigns, etc.; believing that everything happens for a reason and everything must be dealt with patiently; not making comparisons; trying to make a good life in the future; strengthening ones confidence; fighting back; remaining silent; no fear of death, laughing; telling the world what happened; solidarity; trying to ignore bad things; no longer fighting/demonstrating because it is hopeless and costs too much energy.

“Wishes and how the world and others should behave”: Basic human needs have to be fulfilled; being treated like a human being; clarification of the truth; psychosocial and material support; prosecution of perpetrators; political and legal steps; equal treatment in families, communities, and the world; the world has to help!; “We have suffered so much that we are already grateful when someone hands us a glass of water”; everyone can try to teach their children to become good people, so bad people and injustice can be reduced in the world.

## Appendix C. Scoring of the PIQ

For each sub scale as well as the additional scale a score, the mean value is calculated. An overall score is formed as an indicator of the extent to which people perceive injustice. For this purpose, the scale scores (i.e., mean values of each scale) are combined into a sum score which is then divided by the number of scales. Note: the additional items are inverse to the other items. For this reason, the additional score has to be inverted before using it for the overall score:

$$Addtional\;score=-\left(\frac{\left(A1+\;A2+\;A3+\;A4+\;A5\right)}{5}\right)+6$$

$$Overall\;Score=\frac{ECC\;Score+IP\;Score+IE\;Score+RF\;Score+Additional\;Score\;}{5}$$

The following are first proposals for cut-off values. These need to be further researched and validated. In addition, groups need to be identified and specificity and sensitivity need to be explored. These proposals were developed based on the responses of the subgroups.

- Score (ECC) ≥ 3.5
- Score (IP) ≥ 3.5
- Score (IE) ≥ 3.7
- Score (RF) ≥ 3.7

The values of the additional scale can be considered on an item level. The answers should be clarified more closely for all values ≤2. We suggest to set the cut-off value for the overall score at ≤2.3. In other words: people who have an overall score equal or smaller than 2.3 perceive injustice to a higher extent than the average extent. This means that this perception could be considered and might be addressed in their psychotherapy.
